# Alterations in reward network functional connectivity are associated with increased food addiction in obese individuals

**DOI:** 10.1038/s41598-021-83116-0

**Published:** 2021-02-09

**Authors:** Soumya Ravichandran, Ravi R. Bhatt, Bilal Pandit, Vadim Osadchiy, Anita Alaverdyan, Priten Vora, Jean Stains, Bruce Naliboff, Emeran A. Mayer, Arpana Gupta

**Affiliations:** 1 G. Oppenheimer Family Center for Neurobiology of Stress and Resilience, Ingestive Behavior and Obesity Program, CHS 42-210 MC737818, 10833 Le Conte Avenue, Los Angeles, USA; 2 David Geffen School of Medicine At UCLA, Los Angeles, USA; 3 Vatche and Tamar Manoukian Division of Digestive Diseases, Los Angeles, USA; 4 UCLA Microbiome Center, Los Angeles, USA; 5 Ahmanson-Lovelace Brain Mapping Center, University of California Los Angeles (UCLA), Los Angeles, USA; 6 Imaging Genetics Center, Mark and Mary Stevens Neuroimaging and Informatics Institute, University of Southern California, Los Angeles, USA

**Keywords:** Reward, Neuroscience, Insula, Limbic system, Prefrontal cortex, Striatum

## Abstract

Functional neuroimaging studies in obesity have identified alterations in the connectivity within the reward network leading to decreased homeostatic control of ingestive behavior. However, the neural mechanisms underlying sex differences in the prevalence of food addiction in obesity is unknown. The aim of the study was to identify functional connectivity alterations associated with: (1) Food addiction, (2) Sex- differences in food addiction, (3) Ingestive behaviors. 150 participants (females: N = 103, males: N = 47; food addiction: N = 40, no food addiction: N = 110) with high BMI ≥ 25 kg/m^2^ underwent functional resting state MRIs. Participants were administered the Yale Food Addiction Scale (YFAS), to determine diagnostic criteria for food addiction (YFAS Symptom Count ≥ 3 with clinically significant impairment or distress), and completed ingestive behavior questionnaires. Connectivity differences were analyzed using a general linear model in the CONN Toolbox and images were segmented using the Schaefer 400, Harvard–Oxford Subcortical, and Ascending Arousal Network atlases. Significant connectivities and clinical variables were correlated. Statistical significance was corrected for multiple comparisons at q < .05. (1) Individuals with food addiction had greater connectivity between brainstem regions and the orbital frontal gyrus compared to individuals with no food addiction. (2) Females with food addiction had greater connectivity in the salience and emotional regulation networks and lowered connectivity between the default mode network and central executive network compared to males with food addiction. (3) Increased connectivity between regions of the reward network was positively associated with scores on the General Food Cravings Questionnaire-Trait, indicative of greater food cravings in individuals with food addiction. Individuals with food addiction showed greater connectivity between regions of the reward network suggesting dysregulation of the dopaminergic pathway. Additionally, greater connectivity in the locus coeruleus could indicate that the maladaptive food behaviors displayed by individuals with food addiction serve as a coping mechanism in response to pathological anxiety and stress. Sex differences in functional connectivity suggest that females with food addiction engage more in emotional overeating and less cognitive control and homeostatic processing compared to males. These mechanistic pathways may have clinical implications for understanding the sex-dependent variability in response to diet interventions.

## Introduction

As the obesity epidemic progresses, with 42% of the adult U.S. population being obese, rising healthcare costs of over 700 billion dollars annually have been observed^1–3^. Studies have shown associations between obesity and abnormal ingestive behaviors, primarily “food addiction”, in 40% of individuals seeking bariatric surgery^4,5^. Food addiction describes an addictive response in some individuals, where unintended overeating, the increased intake of ultra-processed/hyperpalatable foods beyond homeostatic needs or eating primarily for pleasure occur despite negative consequences^6–8^. The *Yale Food Addiction Scale* (YFAS) is a validated and psychometrically sound measure that uses the DSM-IV diagnostic criteria for substance abuse to operationalize food addiction^9,10^. While it is believed that food addiction is distinct from other behavioral eating disorders, it does share the characteristics or withdrawal, tolerance, impulsivity, and emotional reactivity seen with substance-use disorders and other addictive behaviors^6,7,11^.

Research depicting sex differences in obesity and food addiction have gained momentum to increase treatment efficacy^12^. Although prevalence rates in obesity are similar between the sexes, females report nearly double the rates of food addiction compared to males (12.2–6.4% respectively), with obese females being more likely to encounter loss of control while eating^12,13^. This stems from the increased frequency and cravings towards food as well as the increased reactivity to food cues experienced by females with obesity compared to males^12,14,15^.

Past literature has used magnetic resonance imaging (MRI) as a tool to understand the underlying neurobiology of both obesity and food addiction^16–18^. Individuals with obesity show greater activation in the reward and salience networks, particularly in the basal ganglia, in response to visual stimuli compared to normal weight individuals^19,20^. This has been associated with an increased drive for cravings and greater food consumption, which could explain the prevalence of overeating in obese individuals^21,22^. Studies have also indicated brain connectivity differences related to food addiction between regions of the reward network due to its role in controlling voluntary behavior^23–25^. Altered connectivity in the basal ganglia occurs as a result of the decreased expression of dopamine (D2) receptors which mediate reward-seeking behavior^4,26,27^. The reduction in the availability of D2 receptors creates a perpetual hypodopaminergic state in affected individuals leading to decreasing reward sensitivity, as greater amounts of dopamine must be released to receive the same stimulatory effect^25^. Similar to other addictive behaviors, individuals with food addiction exhibit increased alterations in the regions of the reward network in response to food cues^28–31^. Decreased sensitivity of the reward system translates into decreased inhibitory control and cognitive modulation in food addiction^4,26,27^. Dysregulation of the reward network in conjunction with persistent activation of the dopaminergic pathway supports the compulsive, unregulated food consumption of palatable and high calorie foods seen in obese individuals with food addiction^32,33^.

Sex differences have also been observed in the brain signatures of individuals with obesity. Compared to males, females display an increased reactivity to visual food cues and higher incidences of food cravings, and greater activations in brain regions associated with visual stimuli identification, such as the fusiform gyrus^34^. Additionally, females with obesity showed positive associations between increased BMI and lower connectivity of core reward network regions with cortical and emotion regulation regions^35,36^. This has been linked to the decreased ability to control the physiological response to negative-emotion-inducing stimuli leading to increased emotional eating and food addictive behaviors in females with obesity compared to their male counterparts^37,38^. Sex differences in food addiction have implications for treatments outcomes, as sex differences to naltrexone responsivity have also been observed in other substance abuse disorders, with women generally reporting greater levels of nausea, and poorer treatment adherence and outcomes^39,40^.

Although past literature has addressed the association between food addiction and brain responses to stimuli, few, if any, studies have investigated sex differences in brain signatures, in individuals with and without food addiction. In this study, we aim to examine the neural substrates of food addiction using resting state fMRI and determine sex differences in network connectivity in order to test the following hypotheses: (1) Individuals with food addiction show greater connectivity with regions of the reward network, such as the basal ganglia, compared to individuals with no food addiction. (2) Females with food addiction show greater connectivity with regions of the brainstem, reward, and emotional regulation networks, but lowered connectivity with regions of the brainstem, sensorimotor, and central executive regions compared to males with food addiction. (3) Higher scores on questionnaires measuring altered ingestive eating behaviors are associated with greater connectivity of reward and emotional regulation regions, especially in females.

## Materials and methods

### Subjects

A total of 150 participants (male: N = 47, female: N = 103), ages 18–55 years old, were recruited with the use of flyers and community advertisements and enrolled in the study at the G. Oppenheimer Center for Stress and Resilience. All participants had a BMI greater than 25 kg/m^2^ (obese/overweight; referred to as high BMI henceforth). Participants were excluded for the following: major medical/neurological conditions, current or past psychiatric illness, comorbidities (vascular disease and diabetes), weight loss/abdominal surgeries, pregnancy or breastfeeding, substance use, extreme strenuous exercise (> 8 h of continuous exercise per week), substance use, tobacco dependence (half a pack or more daily), and metal implants. Participants taking medications that interfere with the central nervous system or regular use of analgesic drugs were also excluded. No participants exceeded 400lbs due to magnetic resonance imaging scanning weight limits. Since female sex hormones such as estrogen are known to effect brain structure and function, only premenopausal females were included. All procedures were in compliance with institutional guidelines and were approved by the Institutional Review Board at UCLA’s Office of Protection for Research Subjects. All participants provided written informed consent.

### Questionnaires

Participants were asked to fill out the Yale Food Addiction (YFAS) questionnaire, a 25-item scale developed to measure “food addiction” by assessing signs of substance-dependence symptoms in eating behavior^41^. The Yale Food Addiction Scale (YFAS) has been the commonly utilized measure of food addiction to highly palatable (high fat and high sugar) foods, as these foods have been linked with excess consumption and lowered appetite modulation^4,41^. This scale is based upon the substance dependence criteria found in the DSM-IV^42^ (e.g., tolerance [marked increase in amount; marked decrease in effect], withdrawal [agitation, anxiety, physical symptoms], and loss of control [eating to the point of feeling physical ill])^41^. A symptom count of ≥ 3 together with endorsement of clinically significant impairment or distress on the YFAS denotes diagnostic criteria food addiction (FA). Clinically significant impairment or distress was defined as having a at least one positive response to the following two questions in the YFAS questionnaire: “My behavior with respect to food and eating causes significant distress” and “I experience significant problems in my ability to function effectively (daily routine, job/school, social activities, family activities, health difficulties) because of food and eating,” similar to previously published works^10^. Based on a 353-respondent exploratory survey, the YFAS has displayed a good internal reliability *α* = 0.86^41^. With these measures, participants were placed into one of the following groups based on diagnostic criteria of food addiction (FA): 1. High BMI and with FA (N = 40) 2. High BMI and without FA (N = 110). 3. Females with high BMI and with FA (N = 30), 4. Females with high BMI and without FA (N = 73), 5. Males with high BMI and with FA (N = 10), and 6. Males with high BMI and without FA (N = 37).

Another questionnaire that measures abnormal ingestive behavior was also administered to participants. The General Food Cravings Questionnaire—Traits (GFCQT-r), comprised of 15 items, measures the frequency and intensity of food cravings as a way to study eating patterns and behavior^43^. Higher scores on the GFCQT-r are positively associated with eating pathology, low dieting success, BMI, and increased food cravings^43^. The GFCQT-r has shown high internal consistency (*α* = 0.94) and reliability in its ability to assess food cravings as a trait^44^.

The Hospital Anxiety/Depression Scale (HADS) is a 14-item questionnaire that attempts to identify both possible and probable cases of both anxiety disorders and depression among patients in a non-psychiatric setting^45^. When applied to samples of primary care, psychiatric, and somatic patients, the Hospital Anxiety/Depression Scale has shown strong internal reliability and good concurrent validity^45^.

### Functional magnetic resonance imaging acquisition

Whole brain resting-state functional data was acquired using a 3.0 T Siemens Prisma MRI scanner (Siemens, Erlangen, Germany). Detailed information on the standardized acquisition protocols, quality control measures, and image preprocessing are provided in previously published studies^35,46–50^. Resting-state scans were acquired with eyes closed and an echo planar sequence with the following parameters: TE/TR = 28 ms/2000 ms, flip angle = 77 degrees, scan duration = 8m6s–10m6s, FOV = 220 mm, slices = 40 and slice thickness = 4.0 mm, and slices were obtained with whole-brain coverage. Preprocessing and quality control was done using Statistical Parametric Mapping-12 (SPM12) software and involved bias field correction, co-registration, motion correction, spatial normalization, tissue segmentation, and Fourier transformation for frequency distribution. Data was then spatially normalized to the Montreal Neurological Institute (MNI) template using the structural scans, and then smoothed using a 4 mm isotropic Gaussian kernel.

### Functional network construction

Functional brain networks were constructed as previously described in^35^. To summarize, measures of region-to-region functional connectivity (Fisher transformed Pearson’s correlations) were computed using the CONN toolbox and the aCompCor method in Matlab^51^. Confounding factors such as white matter, cerebrospinal fluid, the six motion realignment parameters, and the root mean squared (RMS) values of the detrended realignment estimates were regressed out for each voxel using ordinary least squares (OLS) regression on the normalized, smoothed resting-state images^52^. Subjects with RMS values over 0.25 were not included. Images were then filtered using a band-pass filter (0.008/s < f < 0.08/s) to reduce the low- and high-frequency noises. Although the influence of head motion cannot be completely removed, CompCor has been shown to be particularly effective for dealing with residual motion relative to other methods^53^. Regions of interest were segmented with the Harvard–Oxford Subcortical atlases, the Schaefer 400 cortical atlas, and the Ascending Arousal Network brainstem atlas^54,55^. These atlases parceled into a total of 430 brain regions. The ROI-ROI functional connectivity between the brain regions was indexed by a matrix of Fisher Z transformed correlation coefficients reflecting the association between average temporal BOLD time series signals across all voxels in each brain region. The magnitude of the Z value represents the weights in the functional network. Permuted statistical values from ROI-to-ROI analyses were further corrected using the false discovery rate (FDR) to measure significance with *p*_(FDR)_ < 0.05.

### Statistical analyses

Descriptive statistical analyses were performed on the 150 subject dataset using SPSS Statistics software on the four groupings listed earlier as well as a combined FA (N = 40) and no FA (N = 110) groups. The General Linear Model (GLM) procedure in SPSS was utilized to analyze the variance and significance between the four groups across all the clinical variables. Second level connectivity analyses were run on CONN to discern sex differences in functional connectivity using GLMs. For all analyses, the disease-dependent analyses (FA vs. no FA) were controlled for age and sex, and all sex difference analyses were controlled for age. The following 5 planned contrasts were used in the statistical analyses for both the clinical variables and connectivity analyses: FA > No FA, Females with FA > Males with FA, Females with FA > Females with no FA, Males with FA > Males with no FA, Females with no FA > Males with no FA.

## Results

### Subject characteristics

All participants had a BMI ≥ 25 kg/m^2^ and were divided into two groups based on diagnostic criteria of FA by their YFAS Symptom Count scores cut off ≥ 3 together with endorsing clinically significant impairment or distress. A complete summary of all the group differences in clinical variables are summarized in Tables 1 and 2.

**Table 1:** Summary of Study Demographics and Clinical Behavioral Measures.

Food addiction (YFAS Diagnostic Score: ≥ 3, and clinical impairment and distress)										No food addiction (YFAS Diagnostic Score: < 3, and low clinical impairment and distress)								
Measurement	Females (N = 30)			Males (N = 10)			Total (N = 40)			Females (N = 73)			Males (N = 37)			Total (N = 110)		
	Mean	SD	N	Mean	SD	N	Mean	SD	N	Mean	SD	N	Mean	SD	N	Mean	SD	N
Age	31.10	10.73	30	28.20	6.29	10	30.35	9.82	40	33.25	9.81	72	33.51	12.38	37	33.34	10.69	110
BMI	31.40	5.13	30	30.11	2.35	10	31.08	4.60	40	31.78	4.46	73	31.36	4.95	37	31.64	4.61	110
**Yale Food Addiction Survey (YFAS)**																		
YFAS withdrawal	0.73	0.94	30	0.90	1.29	10	0.78	1.03	40	0.05	0.23	73	0.08	0.28	37	0.06	0.25	110
YFAS tolerance	0.72	0.80	29	0.14	0.92	10	0.74	0.82	39	0.03	0.17	71	0.06	0.33	36	0.04	0.24	107
YFAS continued use	0.73	0.45	30	0.60	0.52	10	0.70	0.46	40	0.08	0.28	73	0.03	0.16	37	0.06	0.25	110
YFAS given up	0.63	1.16	30	1.30	1.25	10	0.80	1.20	40	0.04	0.26	72	0.00	0.00	37	0.03	0.21	109
YFAS time spent	1.00	0.91	30	0.90	0.88	10	0.98	0.89	40	0.05	0.23	73	0.03	0.16	37	0.05	0.21	110
YFAS loss of control	0.40	0.77	30	0.80	0.92	10	0.50	0.82	40	0.00	0.00	72	0.00	0.00	37	0.00	0.00	109
YFAS unsuccessful cut down	2.30	0.79	30	1.90	0.99	10	2.20	0.85	40	1.43	0.74	68	1.5	0.98	32	1.45	0.82	100
YFAS clinical significant impairment	0.40	0.72	30	0.70	0.82	10	0.48	0.75	40	0.00	0.00	73	0.00	0.00	37	0.00	0.00	110
YFAS symptom count	4.03	1.40	30	4.30	1.42	10	4.10	1.39	40	1.11	0.59	73	0.97	0.55	37	1.06	0.58	110
**General Food Craving Questionnaire (GFCQT)**																		
GFCQT trigger	3.78	1.09	27	4.20	0.84	5	3.84	1.05	32	2.37	1.29	46	2.11	1.13	18	2.30	1.24	64
GFCQT control	16.60	5.32	27	17.6	1.52	5	16.75	4.91	32	9.89	4.64	45	8.72	3.97	18	9.56	4.45	63
GFCQT intentions	7.04	1.99	26	7.40	1.14	5	7.10	1.87	31	4.73	2.45	45	3.83	2.36	18	4.48	2.44	63
GFCQT preoccupation	15.20	5.42	27	16.40	3.72	5	15.41	5.16	32	8.74	4.09	46	7.00	3.11	18	8.25	3.89	64
GFCQT emotions	6.70	2.22	27	7.80	1.30	5	6.88	2.12	32	4.04	1.86	46	3.28	1.84	18	3.83	1.87	64
GFCQT total	49.50	15.36	26	53.40	5.94	5	50.10	14.27	31	29.93	12.92	45	24.94	11.34	18	28.51	12.61	63
**Hospital Anxiety/Depression Scale (HAD)**																		
HAD anxiety	6.26	4.37	23	5.40	3.03	10	6,00	3.98	33	4.39	3.55	72	3.76	3.08	37	4.17	3.39	109
HAD depression	3.17	3.27	23	3.70	3.30	10	3.33	3.24	33	1.97	2.34	72	1.62	1.74	37	1.85	2.15	109

Demonstrates study demographics and clinical behavioral measures for individuals with and without food addiction.

BMI, Body Mass Index; GFCQT, General Food Cravings Questionnaire – Trait; HAD, Hospital Anxiety and Depression Scale; YFAS, Yale Food Addiction Scale; sd, standard deviation; p-value significant < .05.

**Table 2:** Group differences in demographics and clinical behavioral measures.

Measurement	Food addiction versus no food addiction		Females with food addiction versus males with food addiction		Females with food addiction versus females with no food addiction		Males with food addiction versus males with no food addiction	
	F-statistic	p-value	F-statistic	p-value	F-statistic	p-value	F-statistic	p-value
Age	2.92	0.09	0.56	4.00E−03	6.00E−03	6.00E−03	2.01	**0.01**
BMI	9.58	2.00E−03	0.70	0.41	9.62	2.00E−03	3.01	0.09
Height	0.60	0.44	16.33	**8.59E−05**	0.15	0.70	1.29	0.26
Weight	5.23	**0.02**	2.16	0.14	7.07	9.00E−03	1.12	0.29
Hip	2.44	0.12	1.75	0.19	7.45	8.00E−03	0.40	0.84
**Yale Food Addiction Survey (YFAS)**								
YFAS withdrawal	39.76	**3.22E−09**	0.64	0.42	30.21	**1.68E−07**	16.29	**8.72E−05**
YFAS tolerance	53.50	**1.73E−11**	0.20	0.66	45.53	**3.78E−10**	19.72	**1.79E−05**
YFAS continued use	85.23	**2.84E−16**	1.32	0.26	**89.25**	**7.95E−17**	25.59	**1.25E−06**
YFAS given up	51.39	**3.56E−11**	8.34	4.00E−03	18.55	**3.03E−05**	33.29	**4.62E−08**
YFAS time spent	77.77	**3.20E−15**	0.31	0.58	77.79	**3.18E−15**	24.57	**1.96E−06**
YFAS loss of control	48.32	**1.14E−10**	7.02	**0.01**	19.81	**1.69E−05**	29.46	**2.34E−07**
YFAS unsuccessful cut down	13.11	**4.13E−04**	1.74	0.19	23.02	**4.16E−06**	1.77	0.19
YFAS clinical significant impairment	47.65	**1.45E−10**	4.63	**0.03**	23.32	**3.43E−06**	26.44	**8.60E−07**
YFAS symptom count	294.83	**7.44E−37**	0.70	0.40	238.71	**1.60E−32**	114.45	**4.41E−20**
**General Food Craving Questionnaire (GFCQT)**								
GFCQT trigger	27.54	**9.81E−07**	0.53	0.47	23.88	**4.31E−06**	12.09	1.00E−03
GFCQT control	35.85	**4.19E−08**	0.20	0.66	35.25	**5.22E−08**	14.34	**2.74E−04**
GFCQT intentions	21.17	**1.37E−05**	0.11	0.75	17.00	**8.29E−05**	9.67	**0.003**
GFCQT preoccupation	42.61	**3.59E−09**	0.31	0.58	37.97	**1.88E−08**	18.36	**4.50E−05**
GFCQT emotions	43.39	**2.74E−09**	1.34	0.25	31.84	**1.84E−07**	21.16	**1.35E−05**
GFCQT total	42.07	**4.65E−09**	0.38	0.54	36.31	**3.62E−08**	18.31	**4.68E−05**
**Hospital Anxiety/Depression Scale (HAD)**								
HAD anxiety	5.33	**0.02**	0.41	0.52	4.85	**0.03**	1.69	0.12
HAD depression	9.70	2.00E−03	0.32	0.57	4.19	**0.04**	5.65	**0.02**

BMI, Body Mass Index; GFCQT, General Food Cravings Questionnaire—Trait; HAD, Hospital Anxiety and Depression Scale; YFAS, Yale Food Addiction Scale.

p-value significant < .05.

All significant group differences in demographics and clinical behavioral measures for individuals with food addiction (females and males) and individuals with no food addiction (females and males). All bolded values are significant p < 0.05.

Individuals with high BMI and food addiction had higher reported scores on the General Food Craving Questionnaire (GFCQT) (p < 0.05), and higher anxiety (*p* = 0.02) and depression (*p* = 2.00E−3) compared to individuals with no food addiction.

Compared to females with food addiction, males with food addiction had higher scores on the following YFAS subscales: Given Up (*p* = 4.00E−03), Loss of Control (*p* = 9.00E−03), and Clinical Significant Impairment components (*p* = 0.03).

Within females*,* females with food addiction had higher scores on all components of the GFCQT (p < 0.05) and higher anxiety (p = 0.03) and depression (p = 0.04) compared to females without food addiction. Compared to males without food addiction, males with food addiction had higher scores on all components of the GFCQT (p < 0.05), and depression (p = 0.02) scales.

### Food addiction dependent effects on brain connectivity

**Figure 1:**
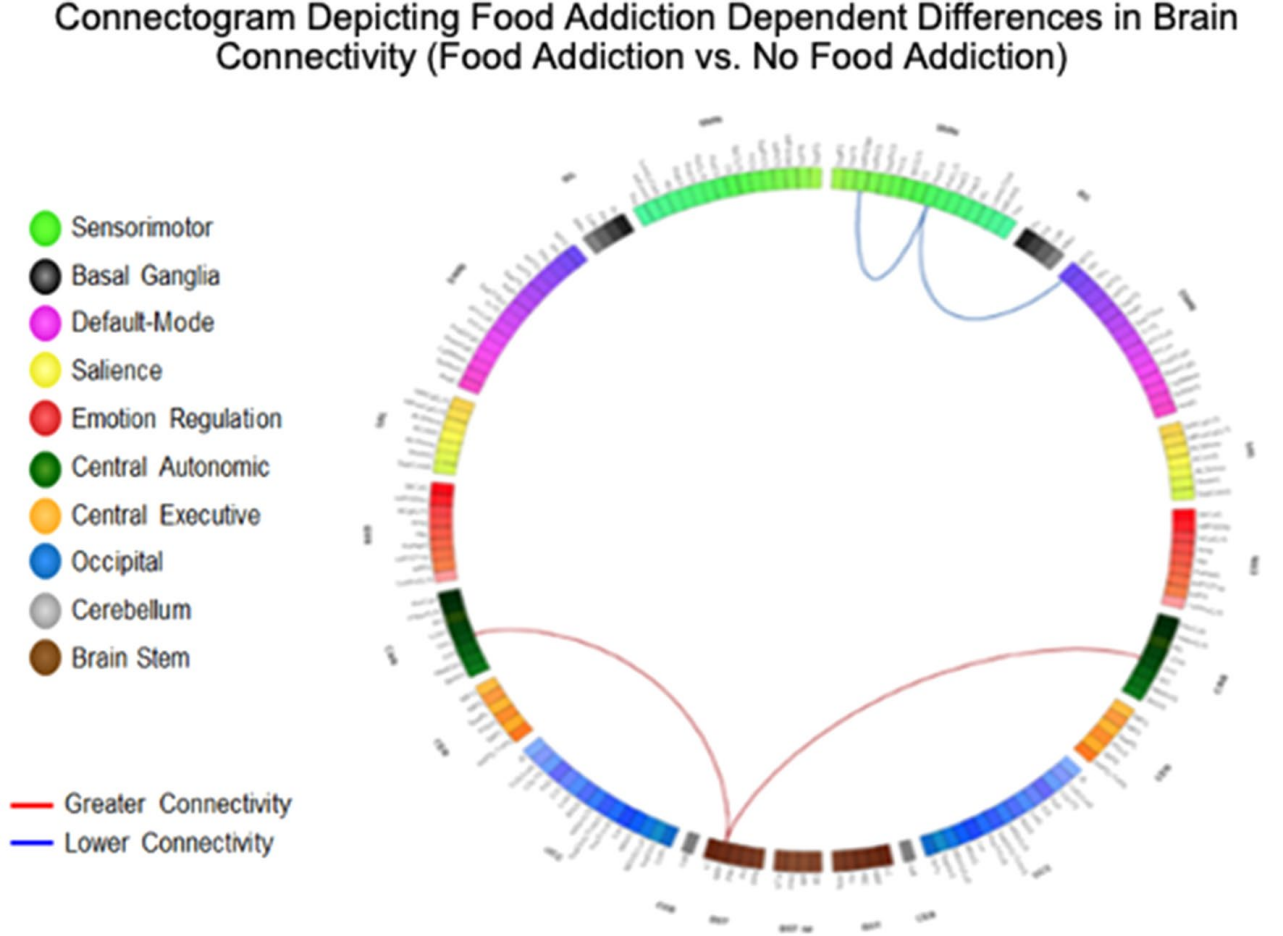
Connectogram depicting food addiction dependent differences in brain connectivity (food addiction vs. no food addiction). Demonstrates significant differences in functional connectivity between individuals with food addiction and individuals with no food addiction. Analysis was performed Harvard–Oxford Subcortical atlases, the Schaefer 400 cortical atlas, and the Ascending Arousal Network brainstem atlas. Labels on the diagram are Destrieux, Harvard–Oxford Subcortical atlases, and the Ascending Arousal Network brainstem atlas equivalents. Red lines between two networks indicate greater functional connectivity, and blue lines indicate lowered functional connectivity. All connections are significant q < 0.05. Light Green: SMN (Sensorimotor Network); Black: BG (Basal Ganglia); Purple: DMN (Default Mode Network); Yellow: SAL (Salience); Red: ERN (Emotional Regulation Network); Dark Green: CAN (Central Autonomic Network); Orange: CEN (Central Executive Network); Blue: OCC (Occipital); Gray: CeB (Cerebellum); Brown: BST (Brain Stem). Bst, Brainstem; CAN, Central Autonomic Network; CEN, Central Executive Network; DMN, Default Mode Network; ERN, Emotional Regulation Network; InfFS, Inferior frontal sulcus; MFG, Middle frontal gyrus; MRF, Mesencephalic reticular formation; MTG, Middle Temporal Gyrus; OrG, Orbital gyri; PosCG, Postcentral Gyrus; SMN, Sensorimotor Network.

Results are detailed in Table 3 and summarized in Table 5, and depicted in Fig. 1.

**Table 3:** Resting state pairwise connections in individuals with food addiction compared to individuals with no food addiction.

Food addiction versus no food addiction								
Network	Analysis unit	Network	Analysis unit	df	t	p-value	q-value	Interpretation
**Brainstem **^113^** connections**								
Bst	L MRF	CEN	L_MFG (R_ContB_PFClv_3)	146	4.05	8.28E−05	0.02	Greater
Bst	L_MRF	CAN	R_OrG, (R_ContB_PFClv_2)	146	3.98	1.08E−04	0.02	Greater
Bst	L_MRF	CAN	L_OrG, (L_ContB_PFClv_1)	146	3.89	1.52E−04	0.02	Greater
**Emotional regulation (ERN) network connections**								
ERN	L_InfFS, (R_ContA_PFCl_2)	SMN	R_PosCG, (R_SomMotA_16)	146	− 4.00	1.00E−04	0.04	Lower
**Sensorimotor (SMN) network connections**								
SMN	R_PosCG, (R_SomMotA_16)	DMN	R_MTG, (R_TempPar_6)	146	− 4.11	6.57E−05	0.03	Lower

Summarizes significant disease-related differences in functional connectivity (individuals with food addiction vs. individuals with no food addiction). All connections are significant q < 0.05.

Bst, Brainstem; CAN, Central Autonomic Network; CEN, Central Executive Network; DMN, Default Mode Network; ERN, Emotional Regulation Network; InfFS, Inferior frontal sulcus; MFG, Middle frontal gyrus; MRF, Mesencephalic reticular formation; MTG, Middle Temporal Gyrus; OrG, Orbital gyri; PosCG, Postcentral Gyrus; SMN, Sensorimotor Network.

df: degrees of freedom; p value significant < .05, q value (corrected for multiple comparisons) < .05.

#### Brainstem connectivity

Individuals with food addiction had greater connectivity between the brainstem and middle frontal gyrus (q = 0.02), and with bilateral orbital gyri (Left and Right q = 0.02) in compared to those without food addiction.

#### Emotional regulation network connectivity

Individuals with food addiction had lowered connectivity between the inferior frontal sulcus and posterior central gyrus compared to those without food addiction (q = 0.04).

#### Sensorimotor network connectivity

Lowered connectivity was found between the postcentral gyrus and middle temporal gyrus in those with food addiction compared to those without food addiction (q = 0.03).

### Sex differences in the food addiction dependent effects on brain connectivity

**Figure 2:**
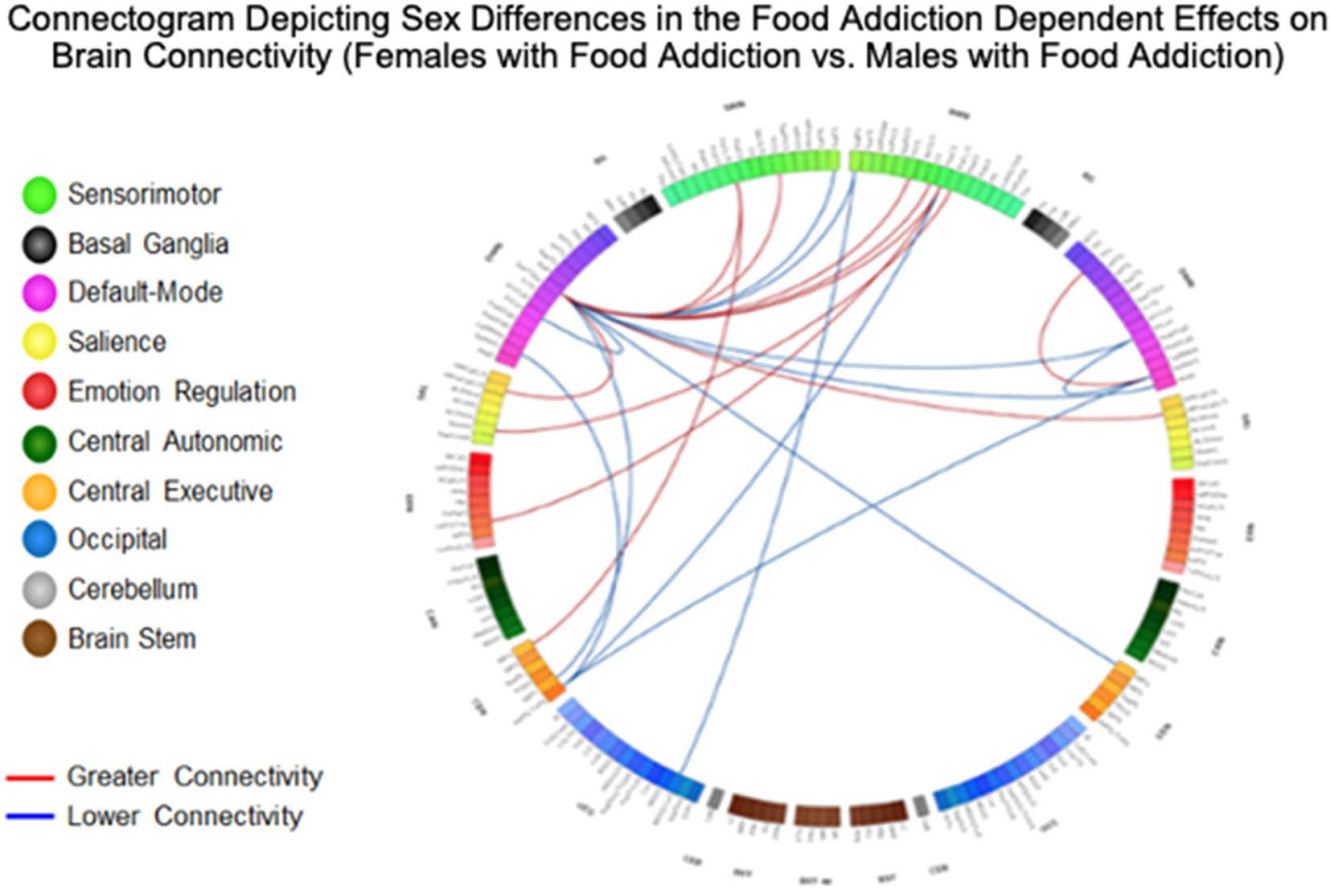
Connectogram Depicting Sex Differences in the Food Addiction Dependent Effects on Brain Connectivity (Females with Food Addiction vs. Males with Food Addiction). Demonstrates significant differences in functional connectivity between females with food addiction and males with food addiction. Analysis was performed Harvard–Oxford Subcortical atlases, the Schaefer 400 cortical atlas, and the Ascending Arousal Network brainstem atlas. Labels on the diagram are Destrieux, Harvard–Oxford Subcortical atlases, and the Ascending Arousal Network brainstem atlas equivalents. Red lines between two networks indicate greater functional connectivity, and blue lines indicate lowered functional connectivity. All connections are significant q < 0.05. Light Green: SMN (Sensorimotor Network); Black: BG (Basal Ganglia); Purple: DMN (Default Mode Network); Yellow: SAL (Salience); Red: ERN (Emotional Regulation Network); Dark Green: CAN (Central Autonomic Network); Orange: CEN (Central Executive Network); Blue: OCC (Occipital); Gray: CeB (Cerebellum); Brown: BST (Brain Stem). AngG, Angular gyrus; CEN, Central Executive Network; CS, Central sulcus (Rolando's fissure); DMN, Default Mode Network; ERN, Emotional Regulation Network; InfFGTrip, Triangular part of the inferior frontal gyrus; IntPS_TrPS, Intraparietal sulcus(interparietal sulcus) and transverse parietal sulci; MFG, Middle frontal gyrus; MOcS_LuS, Middle occipital sulcus and lunatus sulcus; MPosCgG_S, Middle-posterior part of the cingulate gyrus and sulcus; PaCL_S, Paracentral lobule and sulcus; PosCG, Postcentral Gyrus; PRCG, Precentral gyrus; PrCun, Precuneus; ShoInG, Short insular gyri; Tpo, temporal pole; SAL, Salience Network SbPS, Subparietal sulcus; SMN, Sensorimotor Network; SupFG, Superior frontal gyrus; SuMarG, Supramarginal gyrus; SupTGLp, Lateral aspect of the superior temporal gyrus.

Results are detailed in Table 4 and summarized in Table 5, and depicted in Fig. 2.

**Table 4:** Resting state pairwise connections in females with food addiction compared to males with food addiction.

Females with food addiction versus males with food addiction								
Network	Analysis unit	Network	Analysis unit	df	t	p-value	q-value	Interpretation
**Emotional regulation (ERN) network connections**								
ERN	L_InfFGTrip (L_DefaultB_PFCv_4)	SMN	L_PaCL_S (L_SomMotA_6)	147	3.99	1.04E-04	0.04	Greater
**Salience (SAL) network connections**								
SAL	L_ShoInG (L_SalVentAttnA_Ins_1)	SMN	L_PaCL_S (L_SomMotA_13)	147	4.31	2.97E-05	0.01	Greater
SAL	R_MPosCgG_S, (R_SalVentAttnA_ParMed_1)	DMN	L_SupTGLp, (L_TempPar_6)	147	3.64	3.77E-04	0.03	Greater
**Sensorimotor (SMN) network connections**								
SMN	R_SupFG, (R_DefaultA_PFCm_5)	SMN	L_MOcS_LuS, (L_VisCent_ExStr_8)	147	− 3.98	1.08E−04	0.04	Lower
SMN	L_PaCL_S (L_SomMotA_19)	CEN	L_MFG (L_ContB_PFCd_1)	147	4.54	1.16E−05	0.01	Greater
SMN	L_PaCL_S (L_SomMotA_19)	CEN	L_MFG (L_DefaultB_PFCl_2)	147	3.96	1.16E−04	0.03	Greater
SMN	R_PosCG (R_DorsAttnB_PostC_1)	CEN	L_IntPS_TrPS (L_ContB_IPL_3)	147	− 3.66	3.51E−04	0.04	Lower
SMN	L_PRCG, (L_SomMotA_13)	DMN	L_SupTGLp, (L_TempPar_6)	147	3.67	3.39E−04	0.03	Greater
SMN	R_CS, (R_SomMotA_10)	DMN	L_SupTGLp, (L_TempPar_6)	147	3.23	1.53E−03	0.04	Greater
SMN	L_PaCL_S, (L_SomMotA_19)	DMN	L_SupTGLp, (L_TempPar_6)	147	3.50	6.16E−04	0.03	Greater
SMN	R_PRCG, (R_SomMotA_14)	DMN	L_SupTGLp, (L_TempPar_6)	147	3.44	7.57E−04	0.03	Greater
SMN	R_PosCG, (R_SomMotA_6)	DMN	L_SupTGLp, (L_TempPar_6)	147	3.33	1.10E−03	0.04	Greater
SMN	R_SupFG, (R_ContB_PFCmp_1)	DMN	L_SupTGLp, (L_TempPar_6)	147	− 3.98	1.08E−04	0.04	Lower
SMN	L_SupFG, (L_ContB_PFCmp_1)	DMN	L_SupTGLp, (L_TempPar_6)	147	− 3.56	5.00E−04	0.03	Lower
**Central executive network (CEN) connections**								
CEN	R_MFG, (R_ContB_PFCld_4)	DMN	L_SupTGLp, (L_TempPar_6)	147	− 3.63	3.91E−04	0.03	Lower
CEN	L_SbPS, (L_DefaultA_pCunPCC_7)	DMN	L_SupTGLp, (L_TempPar_6)	147	− 3.38	9.28E−04	0.04	Lower
CEN	L_IntPS_TrPS, (L_ContB_IPL_3)	DMN	R_SuMarG, (R_SalVentAttnA_ParOper_2)	147	− 3.96	1.16E−04	0.04	Lower
CEN	L_IntPS_TrPS, (L_ContB_IPL_3)	DMN	R_SuMarG, (R_SalVentAttnA_ParOper_1)	147	− 3.68	3.27E−04	0.04	Lower
CEN	L_IntPS_TrPS, (L_ContB_IPL_3)	DMN	R_SuMarG, (R_SalVentAttnA_ParOper_3)	147	− 3.60	4.34E−04	0.04	Lower
CEN	L_IntPS_TrPS, (L_ContB_IPL_3)	DMN	L_SuMarG, (L_SalVentAttnA_ParOper_1)	147	− 3.63	3.91E−04	0.04	Lower
**Default mode network (DMN) connections**								
DMN	R_SuMarG, (R_TempPar_10)	DMN	R_PrCun, (R_ContC_pCun_3)	147	− 4.37	2.34E−05	0.01	Lower
DMN	R_PrCun, (R_ContC_pCun_3)	DMN	L_SupTGLp, (L_TempPar_6)	147	− 3.70	3.04E−04	0.03	Lower
DMN	L_PrCun, (L_ContC_pCun_3)	DMN	L_SupTGLp, (L_TempPar_6)	147	− 3.62	4.05E−04	0.03	Lower
DMN	R_AngG, (R_ContB_IPL_2)	DMN	L_SupTGLp, (L_TempPar_6)	147	− 3.59	4.50E−04	0.03	Lower
DMN	R_Tpo, (R_LimbicA_TempPole_1)	DMN	R_SuMarG, (R_SalVentAttnA_ParOper_2)	147	3.87	1.63E−04	0.04	Greater
DMN	L_SupTGLp, (L_TempPar_6)	DMN	L_MPosCgG_S, (L_SomMotA_1)	147	3.31	1.17E−03	0.04	Greater

Summarizes significant sex-related differences in functional connectivity (females with food addiction vs. males with food addiction). All connections are significant q < 0.05.

AngG, Angular gyrus; CEN, Central Executive Network; CS, Central sulcus (Rolando's fissure); DMN, Default Mode Network; ERN, Emotional Regulation Network; InfFGTrip, Triangular part of the inferior frontal gyrus; IntPS_TrPS, Intraparietal sulcus(interparietal sulcus) and transverse parietal sulci; MFG, Middle frontal gyrus; MOcS_LuS, Middle occipital sulcus and lunatus sulcus; MPosCgG_S, Middle-posterior part of the cingulate gyrus and sulcus; PaCL_S, Paracentral lobule and sulcus; PosCG, Postcentral Gyrus; PRCG, Precentral gyrus; PrCun, Precuneus; ShoInG, Short insular gyri; Tpo, temporal pole; SAL, Salience Network SbPS, Subparietal sulcus; SMN, Sensorimotor Network; SupFG, Superior frontal gyrus; SuMarG, Supramarginal gyrus; SupTGLp, Lateral aspect of the superior temporal gyrus.

df: degrees of freedom; p value significant < .05, q value (corrected for multiple comparisons) < .05.

**Table 5:** Summary of all group comparisons in network connectivity (food addiction and sex).

Network	Food addiction versus no food addiction	Females with food addiction versus males with food addiction	Females with food addiction versus females with no food addiction	Males with food addiction vs. males with no food addiction
Brainstem	Food addiction ↑		Females with food addiction ↑	Males with food addiction ↑↓
Emotional regulation	Food addiction ↓	Females with food addiction ↑	Females with food addiction ↓	
Salience		Females with food addiction ↑		Males with food addiction ↑
Sensorimotor	Food addiction ↓	Females with food addiction ↑	Females with food addiction ↓	Males with food addiction ↓
Central autonomic			Females with food addiction ↑	
Central executive		Females with food addiction ↓	Females with food addiction ↑	
Default mode		Females with food addiction ↓		Males with food addiction ↑

Summarizes the group (disease effect, sex effect, and within-sex contrasts) network connectivity differences.

↑, Greater connectivity; ↓, Lower Connectivity.

Within sex results detailed in Supplemental Tables S1, S2, and depicted in Supplemental Figures S1, S2.

#### Brainstem connectivity

Compared to females without food addiction, females with food addiction had greater connectivity between the brainstem and middle-anterior part of the cingulate gyrus/sulcus (q = 0.04).

Compared to males without food addiction, males with food addiction had greater connectivity between the brainstem and bilateral middle frontal gyrus, orbital gyrus, and middle occipital gyrus (q = 0.04), but lower connectivity between the brainstem and parahippocampal gyrus (q = 0.03), brainstem and precentral gyrus (q = 0.04), and the brainstem and anterior traverse collateral sulcus (q = 0.04).

#### Emotional regulation network connectivity

Compared to males with food addiction, females with food addiction had greater connectivity between the inferior frontal gyrus and paracentral lobule (q = 0.04).

Compared to females with no food addiction, females with food addiction had lower connectivity between the inferior frontal sulcus and subcentral gyrus (q = 0.01) and between the inferior frontal gyrus and superior parietal lobule (q = 0.03).

#### Salience network connectivity

Compared to males with food addiction, females with food addiction had greater connectivity between the short insular gyrus and paracentral lobule (q = 0.01) and between the middle/posterior part of the cingulate gyrus and superior temporal gyrus (q = 0.03).

Compared to males without food addiction, males with food addiction had greater connectivity between the opercular part of the inferior frontal gyrus and the inferior part of the precentral sulcus (q = 0.03).

#### Sensorimotor network connectivity

Compared to males with food addiction, females with food addiction had greater connectivity between the paracentral lobule/sulcus and the middle frontal gyrus (q = 0.01–0.03), and between the central sulcus, precentral gyrus and superior temporal gyrus (q = 0.03–0.04).

Compared to females without food addiction, females with food addiction had lowered connectivity between the inferior frontal gyrus and subcentral gyrus (q = 0.02) and between the precentral gyrus and lingual gyrus (q = 0.04).

Compared to males with no food addiction, males with food addiction had lowered connectivity between the paracentral lobule and middle frontal gyrus (q = 0.04).

#### Central autonomic network connectivity

Compared to females without food addiction, females with food addiction had greater connectivity between the anterior cingulate gyrus and sulcus and paracentral lobule (q = 3.00E-03).

#### Central executive network connectivity

Compared to males with food addiction, females with food addiction had lowered connectivity between the middle frontal gyrus and superior temporal gyrus (q = 0.03), intraparietal sulcus and supramarginal gyrus (q = 0.04), and superior frontal gyrus and superior temporal gyrus (q = 0.03–0.04).

Compared to females without food addiction, females with food addiction had greater connectivity between the intraparietal sulcus and orbital gyrus (q = 0.03).

#### Default mode network connectivity

Compared to males with food addiction, females with food addiction had lowered connectivity between the supramarginal gyrus and the precuneus (q = 0.01), precuneus and superior temporal gyrus (q = 0.03), and superior temporal gyrus and angular gyrus (q = 0.03).

Compared to males without food addiction, males with food addiction had greater connectivity between the supramarginal gyrus and the precuneus (q = 0.02) and between the temporal pole and orbital sulcus (q = 0.03).

### Associations between brain connectivity and clinical variables

#### Food addiction associations

Results are summarized in Table 6.

**Table 6:** Associations between significant functional connectivities and clinical variables.

Food addiction versus no food addiction					
**Food addiction**					
No significant results					
**No food addiction**					
Functional Connectivity	Clinical variables	r	p	q	df
MRF to right OrG*	BMI	0.35	2.10E−04	0.01	106
MRF to left OrG	GFCQT intentions	− 0.38	2.00E−03	**0.04**	59
**Females with food addiction versus males with food addiction**					
**Females with food addiction**					
No significant results					
**Males with food addiction**					
No significant results					
**Females with food addiction vs females with no food addiction**					
**Females with food addiction**					
Functional connectivity	Clinical variables	r	p	q	df
LC to left ACgG_S	BMI	0.61	4.00E-04	0.04	27
**Females with no food addiction**					
No significant results					
**Males with food addiction versus males with no food addiction**					
**Males with food addiction**					
No significant results					
**Males with no food addiction**					
No significant results					

Summarizes correlations between functional connectivity and clinical variables. Comparisons include disease differences (individuals with food addiction vs. individuals with no food addiction), sex differences (females with food addiction vs. males with food addiction), disease effect within females (females with food addiction vs. females with no food addiction), and disease effect within males (males with food addiction vs. males with no food addiction). All connections are significant q < 0.05.

*Right ContB_PFClv_3.

ACgG_S, Middle-anterior part of thecingulate gyrus and sulcus; BMI, Body Mass Index; GFCQT, General Food Cravings Questionaire—Trait; LC, locus coeruleus; MRF, Mesencephalic reticular formation; OrG, Orbital gyri.

r: correlation, df: degrees of freedom; p value significant < .05, q value (corrected for multiple comparisons) < .05.

For individuals with no food addiction, connectivity between the brainstem (mesencephalic reticular formation) and central autonomic network (orbital gyrus) was positively associated with BMI (r = 0.35, q = 0.01) and negatively associated with the Intention component of the GFCQT (r = − 0.38, q = 0.04).

#### Sex difference associations

Results are summarized in Table 6.

In females with food addiction, connectivity between the brainstem (locus coeruleus) and emotional regulation network (middle-anterior part of the cingulate gyrus and sulcus) was positively associated with BMI (r = 0.61, q = 0.04).

## Discussion

The goal of this study was to identify sex-related differences in the connectivity of brain networks in individuals meeting diagnostic criteria for food addiction. The main findings of the study were: 1) Food addiction was associated with greater connectivity among the reward regions and between the brainstem and central autonomic networks, and lower connectivity among the emotional regulation, sensorimotor, and default mode networks compared to those with no food addiction. 2. Sex differences were observed with females showing greater connectivity in the emotional regulation and salience networks and lower connectivity in the brainstem, central executive, and default mode networks. Our results support the hypothesis that altered connectivity in reward regions could increase the risk for addictive ingestive behaviors, resulting in the uncontrollable overeating patterns seen in individuals with clinically significant impairment or distress with food addiction. Additionally, our results indicate greater connectivity in particular resting-state networks, such as the emotional regulation and salience networks, which could explain the higher rates of emotional eating and food addiction seen in females. To our knowledge, this is the first study to investigate sex-related differences in resting-state connectivity in individuals with food addiction.

### Food addiction dependent effects on brain connectivity

Uncontrollable eating seen in food addiction, can be explained by the reward deficiency hypothesis which states that a decreased availability of dopamine receptors, specifically D2 receptors, creates a less responsive reward system that is susceptible to addictive pathologies^35,56–60^. Drug-addiction studies have associated drug-dependence with changes in the dopamine receptor availability, with individuals with decreased receptors seeking greater and more frequent reward stimulation^57^. In individuals with food addiction, high sugar/high fat foods act as this source of stimulation, as such these ultra-processed foods serve as potent reward triggers that increase synaptic dopamine concentration overriding internal satiety cues^57,61^. Hence, the constant hypodopaminergic state of these individuals results in increased levels of food cravings and overindulgence of ultra-processed foods as a compensatory attempt to derive the euphoric effects of reward network stimulation^62,63^. Studies have also suggested that disruptions in the mesolimbic pathways associated with addiction behaviors effects both the DA reward circuits and DA pathways that lead to increased stress reactivity and disruption in interoceptive awareness^29,57,64^.

Consistent with the reward deficiency hypothesis, our results indicated greater connectivity between the brainstem and reward regions in individuals with food addiction. This could be associated with dopaminergic dysregulation, as the increased intake of ultra-processed foods, in those with food addiction leads to a more persistent stimulation of the reward pathway as a compensatory mechanism for the decreased receptor sensitivity and availability^19,33,65^. On the other hand, in those with no food addiction, a greater connectivity between the brainstem and the central autonomic network (orbital gyrus) was negatively associated with GFCQT scores, a measure of food cravings. Perhaps, in individuals with food addiction, increases in brainstem connectivity could be a result of an increased frequency of dopamine release in response to select salient inputs, such as ultra-processed foods, causing heightened reactions and cravings to consume larger quantities of such foods^66,67^. This increased brainstem connectivity, could be a counterbalancing mechanism for the reduction in dopamine receptors in the reward circuit per the reward deficiency hypothesis^26,32,57,68^.

In addition to decreased reward sensitivity, hyperactivation of the locus coeruleus could also contribute to the habitual overconsumption of ultra-processed foods seen in individuals with food addiction. The locus coeruleus plays a major role in an individual’s response to external stressors, with greater activation being linked to consistent higher levels of norepinephrine seen in individuals with anxiety^69^. Individuals in a sustained state of anxiety, consistently have elevated levels of norepinephrine which result in cortical atrophy leading to reduced cognitive and attentional control^70^. Thus, greater activation of the locus coeruleus could translate to a greater susceptibility to maladaptive, habitual behaviors such as food addiction due a combination of decreased internal regulation capabilities and perpetual anxiety in individuals with food addiction^69,70^.

Our results mirrored these predicted differences, with individuals with food addiction displaying significantly higher rates of anxiety, particularly in females. Additionally, greater connectivity between the locus coeruleus and the emotional regulation network was positively associated with BMI in females with food addiction. These results suggest the role of the locus coeruleus in weight management may contribute to the food addictive behaviors in an attempt to cope with anxiety.

### Sex differences in the food addiction dependent effects on brain connectivity

Compared to males with food addiction, females with food addiction displayed significantly higher rates of emotional overeating^37,38^. Emotional overeating is related to cognitive alterations in two aspects: the inability to regulate emotional states and the inability to limit consumption of especially ultra-processed foods when in a compromised emotional state^71^. According to the self-medication hypothesis, individuals with difficulties in self-regulation turn to specific actions or external substances to help relieve the negative emotional impact of stimuli, resulting in substance abuse disorders^72,73^. In the case of food addiction, females with food addiction utilize high sugar/high fat foods as their sources of stimulation, in an attempt to lessen their emotional load^57^. The second aspect of emotional overeating, the reduced capability to limit intake of ultra-processed foods, is a result of lowered cognitive inhibitions created by heightened reactivity to food cues and attenuated satiety responses^74–76^ .

According to the incentive salience model, the motivational value of a “reward” is based on trigger cues, such as the sight, smell, and taste of ultra-processed foods^77^. In cases of addiction, other rewards are perceived to have a diminished incentive value relative to the drug causing compulsive behavior, reorientation of attentional resources, and downregulation of cognitive control regions^57,78^. This creates an increased reward being placed on ultra-processed foods, resulting in individuals seeking out these types of food beyond their basic homeostatic needs and even when their consumption leads to negative consequences^57^. In addition, due to the chronic consumption of high sugar/high fat foods, satiety signals in these individuals are compromised, as the palatability of ultra-processed food stimuli overrides an individual’s energy need^79^. These altered satiety cues in conjunction with increased response to food cues translate into more frequent and greater overeating behaviors.

Our results support this emotional overeating model, with females with food addiction exhibiting greater connectivity between the emotional regulation and salience networks compared to males with food addiction. Greater activation of the emotional regulation network aligns with the self-medication hypothesis, as females with food addiction engage in uncontrollable eating behaviors as an artificial coping mechanism to manage their emotional response to negative stimuli, similar to that of a drug^46,80–84^. The tendency of females with food addiction to actively seek out and consume ultra-processed foods in response to negative emotional stimuli, could refer to the relationship between greater emotional instability and increased food-seeking behavior as predicted in our model^67,85^.

The greater connectivity observed in the salience network, particularly between the salience and default mode network, is similarly consistent with our hypothesis and the incentive salience model^77,86^. The salience network adjusts attentional resources to salient sensory stimuli, with the observed greater activation suggesting an increased attentional value on food cues^12,81,87–90^. As females with food addiction place greater value and attention towards ultra-processed foods, the “incentive value” of this stimuli increases, resulting in a greater subconscious food focus and food cravings^77,86,91^. Additionally, this greater preoccupation towards ultra-processed foods, seen in females with food addiction, may also cause inhibition of cognitive regions in areas of the default-mode network leading to increased decisional impulsivity and lowered inhibitory responses in response to salient stimuli^92,93^.

Compared to males with food addiction, females with food addiction had greater connectivity among the sensorimotor network, particularly in the regions controlling evaluation of external stimuli^94–97^. These regions, namely the precentral gyrus and paracentral lobule, have been linked with altered explicit memory and inappropriate cognitive evaluations, potentially resulting in an increased motivational reward placed on food-related stimuli in females with food addiction^98,99^.

Additionally, when compared to females with no food addiction, those with food addiction exhibited lower connectivity in the sensorimotor network in regions involved in inhibition and attentional control, such as the inferior frontal gyrus^100^. Hypoactivation in the inferior frontal gyrus suggests increased impulsivity and the reinforcement of habit-forming systems, leading to weakened attempts to disengage with compulsive eating behaviors^101^.

Consistent with our model, females with food addiction had lowered connectivity within the default mode network compared to males with food addiction, potentially explaining the reduced cognitive control and greater preoccupation with food-related stimuli predicted in females with food addiction^102^. The default mode network plays a major role in self-generated and subconscious thought, displaying greater activation when resting^103,104^. Specifically, the dorsal medial prefrontal cortex subsystem has shown to preferentially activate during subconscious decision-making processes regarding one’s present mental state or situation^105,106^. Lower activation in this subsystem could translate to decreased subconscious inhibitions and altered attentional processing as a result of the top-down inhibition on cognitive control regions^102,107,108^. Weakened cognitive control circuits, upon interacting with the reward and emotional regulation system, reorient attentional resources in those with food addiction and create a vicious cycle of chronic overconsumption, impaired appetite regulation, and heightened food focus^109^. Additionally, consistent with the incentive salience model, our results showed lowered connectivity between the default mode network and the central executive network in females with food addiction, indicating that the lowered awareness of the present state results in less sensitive internal body cues and chronic food consumption behaviors as predicted in our hypothesis^74–76^.

### Limitations and future research

Due to the cross-sectional design of the study, we were unable to address questions of causality between functional connectivity differences and food addiction. Future longitudinal studies are needed to determine if the observed connectivity differences between brain networks in individuals with food addiction are a premorbid state, or if they are a consequence of food addiction and associated metabolic changes. The integration of systemic inflammatory markers and metabolites derived from gut microbiota as mediators can help gain a more comprehensive understanding of food addiction in future mechanistic studies. While this study focused on resting-state connectivity differences, future research should consider investigating connectivity differences in response to ultra-processed food cues in individuals with food addiction. In order to be able to combine data to obtain a large sample size for subgroup analyses, all participants in this study completed the earlier version of the YFAS vs. the updated YFAS 2.0 questionnaire which is not only more psychometrically sound but has a stronger threshold of FA associated with obesity. This may have contributed to a reduced sensitivity in observing significant results, which will need to be validated in future studies using the YFAS 2.0.

### Conclusions and clinical implications

When viewed together with previous findings, our results show greater connectivity in reward regions, indicative of the altered function of the reward circuit in individuals with food addiction. Sex differences in functional connectivity reveal that females with food addiction engage in more emotional eating behaviors while males with food addiction exhibit greater cognitive control and homeostatic processing. Since connectivity differences differ among males and females, this study contributes to the understanding of the nuances driving the sex-specific pathophysiology of food addiction. These mechanistic pathways may have clinical implications for understanding the variability in response to diet interventions and the need for more effective, targeted treatments for those with food addiction, especially females. Most clinical trials do not report sex differences related to treatment responses, but a few existing reports suggest that women are less likely to complete treatment, tend to lose less weight than men, have greater unsuccessful attempts to maintain weight loss resulting in the well-known “YoYo” diet phenomenon, and have limited responses to pharmacological treatments^12,110,111^. Therefore, treatments need to focus on the different sex-related eating patterns, such as women gaining weight via eating ultra-processed foods more frequently during emotional or stressful times, as compared to men, who gain weight via the consumption of larger meals. Therapeutic approaches would need to target different nodes of the brain related to food addiction, and individualization of treatments would need to be based on sex-related differences in order to improve greater clinical benefits^13,112^.

## Supplementary Information

Supplementary Information 1.
