# Supplementary material for: Alterations in reward network functional connectivity are associated with increased food addiction in obese individuals
Source: Sci Rep. 2021 Feb 9;11:3386. doi: 10.1038/s41598-021-83116-0 (PMC7873272; doi:10.1038/s41598-021-83116-0)

Alterations in Reward Network Functional Connectivity are Associated with Increased Food Addiction in Obese Individuals

Soumya Ravichandran[1], Ravi R. Bhatt[1,6], Bilal Pandit[1], Vadim Osadchiy[1,2], Anita Alaverdyan[1], Priten Vora[1], Jean Stains[1-3], Bruce Naliboff[1-4], Emeran A. Mayer[1-5], Arpana Gupta*[1-4]

SUPPLEMENTARY MATERIALS

SUPPLEMENTARY TABLES

Supplemental Table S1. Resting State Pairwise Connections Comparing Females with Food Addiction vs. Females with No Food Addiction

This table summarizes significant functional connectivity, comparing disease effect within females (females with food addiction vs. females with no food addiction). All connections are significant q<0.05.

Abbreviations: ACgG_S, Middle-anterior part of the cingulate gyrus and sulcus; Bst, Brainstem Network; CAN, Central Autonomic Network; CEN, Central Executive Network; ERN, Emotional Regulation Network; InfFGOpp, Opercular part of the inferior frontal gyrus; InfFGTrip, Triangular part of the inferior frontal gyrus; InfFS, Inferior frontal sulcus; IntPS_TrPS, Intraparietal sulcus(interparietal sulcus) and transverse parietal sulci; LC, locus coeruleus; LinG, Lingual gyrus, lingual part of the medial occipito-temporal gyrus; OCC, Occipital Network; OrG, Orbital gyri; PaCL_S, Paracentral lobule and sulcus; PRCG, Precentral gyrus; SbCG_S, Subcentral gyrus and sulci; SMN, Sensorimotor Network; SupPL, Superior parietal lobule(lateral part of P1)

df: degrees of freedom; p value significant <.05, q value (corrected for multiple comparisons) <.05

Supplemental Table S2. Resting State Pairwise Connections Comparing Males with Food Addiction vs. Males with No Food Addiction

This table summarizes significant functional connectivity, comparing disease effect within males (males with food addiction vs. males with no food addiction). All connections are significant q<0.05.

Abbreviations: ATrCoS, Anterior transverse collateral sulcus; Bst, Brainstem Network; CAN, Central Autonomic Network; CEN, Central Executive Network; CS, Central sulcus(Rolando's fissure); DMN, Default Mode Network; ERN, Emotional Regulation Network; InfFGOpp, Opercular part of the inferior frontal gyrus; InfPrCS, Inferior part of the precentral sulcus; MOcG, Middle occipital gyrus; MFG, Middle frontal gyrus; MR, Median raphé nuclei; MRF, Mesencephalic reticular formation; OCC, Occipital Network; OrG, Orbital gyri; OrS, Orbital sulci(H-shaped sulci); PaCL_S, Paracentral lobule and sulcus; PaHipG, Parahippocampal gyrus, parahippocampal part of the medial occipito-temporal gyrus; PBC, Parabrachial complex; PRCG, Precentral gyrus; PrCun, Precuneus; Tpo, temporal pole; SAL, Salience Network; SMN, Sensorimotor Network; SuMarG, Supramarginal gyrus

df: degrees of freedom; p value significant <.05, q value (corrected for multiple comparisons) <.05

SUPPLEMENTARY FIGURES

Supplemental Figure S1. Connectogram Depicting Differences in Brain Connectivity *between* Females with Food Addiction vs. Females with No Food Addiction

Supplemental Figure 1 is a connectogram demonstrating significant differences in functional connectivity between females with food addiction and females with no food addiction. Analysis was performed Harvard-Oxford Subcortical atlases, the Schaefer 400 cortical atlas, and the Ascending Arousal Network brainstem atlas. Labels on the diagram are Destrieux, Harvard-Oxford Subcortical atlases, and the Ascending Arousal Network brainstem atlas equivalents. Red lines between two networks indicate greater functional connectivity, and blue lines indicate lowered functional connectivity. All connections are significant q<0.05.

Legend: Light Green: SMN (Sensorimotor Network); Black: BG (Basal Ganglia); Purple: DMN (Default Mode Network); Yellow: SAL (Salience); Red: ERN (Emotional Regulation Network); Dark Green: CAN (Central Autonomic Network); Orange: CEN (Central Executive Network); Blue: OCC (Occipital); Gray: CeB (Cerebellum); Brown: BST (Brain Stem)

Abbreviations: ACgG_S, Middle-anterior part of the cingulate gyrus and sulcus; Bst, Brainstem Network; CAN, Central Autonomic Network; CEN, Central Executive Network; ERN, Emotional Regulation Network; InfFGOpp, Opercular part of the inferior frontal gyrus; InfFGTrip, Triangular part of the inferior frontal gyrus; InfFS, Inferior frontal sulcus; IntPS_TrPS, Intraparietal sulcus(interparietal sulcus) and transverse parietal sulci; LC, locus coeruleus; LinG, Lingual gyrus, lingual part of the medial occipito-temporal gyrus; OCC, Occipital Network; OrG, Orbital gyri; PaCL_S, Paracentral lobule and sulcus; PRCG, Precentral gyrus; SbCG_S, Subcentral gyrus and sulci; SMN, Sensorimotor Network; SupPL, Superior parietal lobule(lateral part of P1)

Supplemental Figure S2. Connectogram Depicting Differences in Brain Connectivity *between* Males with Food Addiction vs. Males with No Food Addiction

Supplemental Figure 2 is a connectogram demonstrating significant differences in functional connectivity between males with food addiction and males with no food addiction. Analysis was performed Harvard-Oxford Subcortical atlases, the Schaefer 400 cortical atlas, and the Ascending Arousal Network brainstem atlas. Labels on the diagram are Destrieux, Harvard-Oxford Subcortical atlases, and the Ascending Arousal Network brainstem atlas equivalents. Red lines between two networks indicate greater functional connectivity, and blue lines indicate lowered functional connectivity. All connections are significant q<0.05.

Legend: Light Green: SMN (Sensorimotor Network); Black: BG (Basal Ganglia); Purple: DMN (Default Mode Network); Yellow: SAL (Salience); Red: ERN (Emotional Regulation Network); Dark Green: CAN (Central Autonomic Network); Orange: CEN (Central Executive Network); Blue: OCC (Occipital); Gray: CeB (Cerebellum); Brown: BST (Brain Stem)

Abbreviations: ATrCoS, Anterior transverse collateral sulcus; Bst, Brainstem Network; CAN, Central Autonomic Network; CEN, Central Executive Network; CS, Central sulcus(Rolando's fissure); DMN, Default Mode Network; ERN, Emotional Regulation Network; InfFGOpp,

Opercular part of the inferior frontal gyrus; InfPrCS, Inferior part of theprecentral sulcus; MOcG, Middle occipital gyrus; MFG, Middle frontal gyrus; MR, Median raphé nuclei; MRF, Mesencephalic reticular formation; OCC, Occipital Network; OrG, Orbital gyri; OrS, Orbital sulci(H-shaped sulci); PaCL_S, Paracentral lobule and sulcus; PaHipG, Parahippocampal gyrus, parahippocampal part of the medial occipito-temporal gyrus; PBC, Parabrachial complex; PRCG, Precentral gyrus; PrCun, Precuneus; Tpo, temporal pole; SMN, Sensorimotor Network; SuMarG, Supramarginal gyrus

Supplemental Table S1. Resting State Pairwise Connections Comparing Females with Food Addiction vs. Females with No Food Addiction

FEMALES WITH FOOD ADDICTION vs FEMALES WITH NO FOOD ADDICTION								
**Network**	**Analysis Unit**	**Network**	**Analysis Unit**	**df**	**t**	**p-value**	**q-value**	**Interpretation**
**Brainstem [97] Connections**								
Bst	LC (locus coeruleus)	ERN	Right ACgG_S (Right DefaultA_PFCm_1)	147	3.81	2.04E-04	0.04	Greater
Bst	LC (locus coeruleus)	ERN	Left ACgG_S (Left DefaultA_PFCm_4)	147	3.80	2.11E-04	0.04	Greater
**Emotional Regulation (ERN) Connections**								
ERN	Left InfFS (Left ContA_PFCLeft 2)	SMN	Left SbCG_S (Left SomMotB_S2_5)	147	-4.33	2.74E-05	0.01	Lower
ERN	Right InfFGTrip (Right ContA_PFCLeft 1)	SMN	Left PaCL_S (Left SomMotA_14)	147	-3.89	1.51E-04	0.03	Lower
ERN	Right InfFGTrip (Right ContA_PFCLeft 1)	CEN	Left SupPL (Left DorsAttnB_PostC_9)	147	-4.09	7.08E-05	0.03	Lower
**Sensorimotor Network (SMN) Connections**								
SMN	Right InfFGOpp (Right ContA_PFCLeft 2)	SMN	Left SbCG_S (Left SomMotB_S2_5)	147	-4.26	3.63E-05	0.02	Lower
SMN	Left PRCG (Left SomMotA_9)	OCC	Left LinG (Left VisCent_Striate_1)	147	-3.97	1.12E-04	0.04	Lower
**Central Autonomic Network (CAN) Connections**								
CAN	Left ACgG_S (Left DefaultA_PFCm_2)	SMN	Left PaCL_S (Left SomMotA_19)	147	4.66	7.03E-06	3.00E-03	Greater
**Central Executive Network (CEN) Connections**								
CEN	Right IntPS_TrPS (Right DorsAttnA_SPLeft 4)	CAN	Left OrG (Left ContB_PFClv_1)	147	4.10	6.81E-05	0.03	Greater

Abbreviations: ACgG_S, Middle-anterior part of thecingulate gyrus and sulcus; Bst, Brainstem Network; CAN, Central Autonomic Network; CEN, Central Executive Network; ERN, Emotional Regulation Network; InfFGOpp, Opercular part of the inferior frontal gyrus; InfFGTrip, Triangular part of the inferior frontal gyrus; InfFS, Inferior frontal sulcus; IntPS_TrPS, Intraparietal sulcus(interparietal sulcus) and transverse parietal sulci; LC, locus coeruleus; LinG, Lingual gyrus, lingual part of the medial occipito-temporal gyrus; OCC, Occipital Network; OrG, Orbital gyri; PaCL_S, Paracentral lobule and sulcus; PRCG, Precentral gyrus; SbCG_S, Subcentral gyrus and sulci; SMN, Sensorimotor Network; SupPL, Superior parietal lobule(lateral part of P1)

df: degrees of freedom; p-value sig <.05, q-value (corrected for multiple comparisons) <.05

Supplemental Table S2. Resting State Pairwise Connections Comparing Males with Food Addiction vs. Males with No Food Addiction

MALES WITH FOOD ADDICTION vs MALES WITH NO FOOD ADDICTION								
**Network**	**Analysis Unit**	**Network**	**Analysis Unit**	**df**	**t**	**p**	**q**	**Interpretation**
**Brainstem [97] Connections**								
Bst	MRF	CEN	Right MFG (Right ContB_PFCld_4)	147	3.66	3.51E-04	0.04	Greater
Bst	MRF	CEN	Right MFG (Right SalVentAttnB_PFCLeft 2)	147	3.52	5.75E-04	0.04	Greater
Bst	MRF	CEN	Right MFG (Right SalVentAttnB_PFCLeft 3)	147	3.45	7.32E-04	0.04	Greater
Bst	MRF	CEN	Left MFG (Left DefaultB_PFCLeft 1)	147	3.64	3.77E-04	0.04	Greater
Bst	MRF	CAN	Left OrG (Left ContB_PFClv_1)	147	3.39	8.98E-04	0.04	Greater
Bst	MRF	CAN	Left OrG (Left ContB_PFClv_1)	147	3.39	8.98E-04	0.04	Greater
Bst	MRF	OCC	Right MOcG (Right DefaultC_IPLeft 2)	147	3.57	4.83E-04	0.04	Greater
Bst	MRF	SMN	Right PRCG (Right SomMotA_7)	147	-3.41	8.39E-04	0.04	Lower
Bst	MRF	SMN	Right CS (Right SomMotA_10)	147	-3.41	8.39E-04	0.04	Lower
Bst	MR	DMN	Left ATrCoS (Left LimbicA_TempPole_5)	147	-4.04	8.58E-05	0.04	Lower
Bst	PBC	ERN	Left PaHipG (Left LimbicA_TempPole_3)	147	-4.07	7.65E-05	0.03	Lower
**Salience Network (SAL) Connections**								
SAL	Left InfFGOpp (Left SalVentAttnA_FrOpeRight 1)	SMN	Left InfPrCS (Left ContA_PFCLeft 1)	147	4.08	7.36E-05	0.03	Greater
**Sensorimotor Network (SMN) Connections**								
SMN	Left PaCL_S (Left SomMotA_19)	CEN	Left MFG (Left ContB_PFCd_1)	147	-4.00	1.00E-04	0.04	Lower
**Default Mode Network (DMN) Connections**								

DMN	Right SuMarG (Right TempPaRight 10)	DMN	Right PrCun (Right ContC_pCun_3)	147	4.15	5.61E-05	0.02	Greater
DMN	Left Tpo (Left LimbicA_TempPole_1)	CAN	Right OrS (Right LimbicB_OFC_2)	147	4.07	7.65E-05	0.03	Greater

<u>Abbreviations</u>: ATrCoS, Anterior transverse collateral sulcus; Bst, Brainstem Network; CAN, Central Autonomic Network; CEN, Central Executive Network; CS, Central sulcus(Rolando's fissure); DMN, Default Mode Network; ERN, Emotional Regulation Network; InfFGOpp, Opercular part of the inferior frontal gyrus; InfPrCS, Inferior part of theprecentral sulcus; MOcG, Middle occipital gyrus; MFG, Middle frontal gyrus; MR, Median raphé nuclei; MRF, Mesencephalic reticular formation; OCC, Occipital Network; OrG, Orbital gyri; OrS, Orbital sulci(H-shaped sulci); PaCL_S, Paracentral lobule and sulcus; PaHipG, Parahippocampal gyrus, parahippocampal part of the medial occipito-temporal gyrus; PBC, Parabrachial complex; PRCG, Precentral gyrus; PrCun, Precuneus; Tpo, temporal pole; SAL, Salience Network; SMN, Sensorimotor Network; SuMarG, Supramarginal gyrus

df: degrees of freedom; p value significant <.05, q value (corrected for multiple comparisons) <.05

Connectogram Depicting Differences in Brain Connectivity *between* Females with Food Addiction vs. Females with no Food Addiction

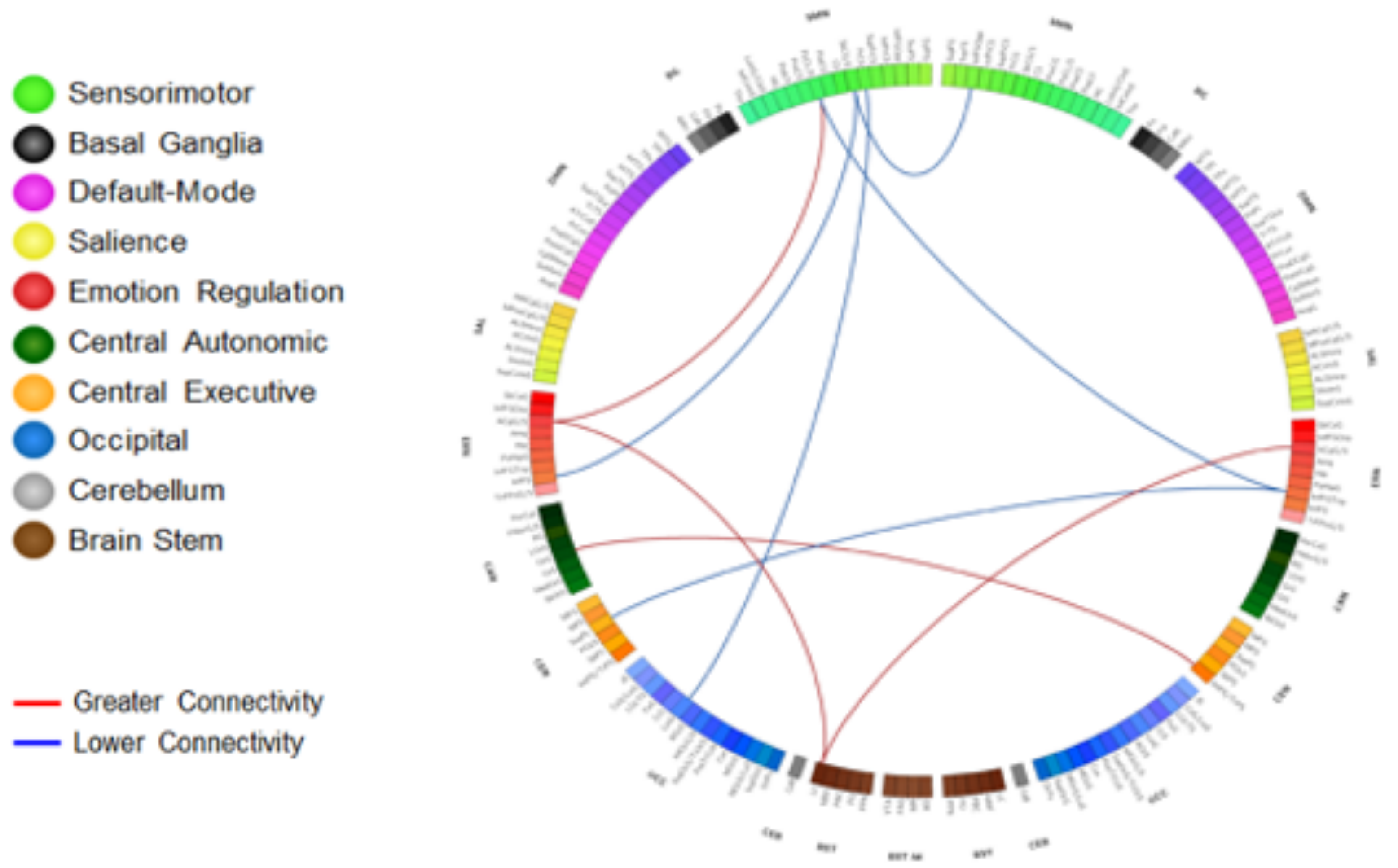

Connectogram Depicting Differences Brain Connectivity *between* Males with Food Addiction vs. Males with no Food Addiction

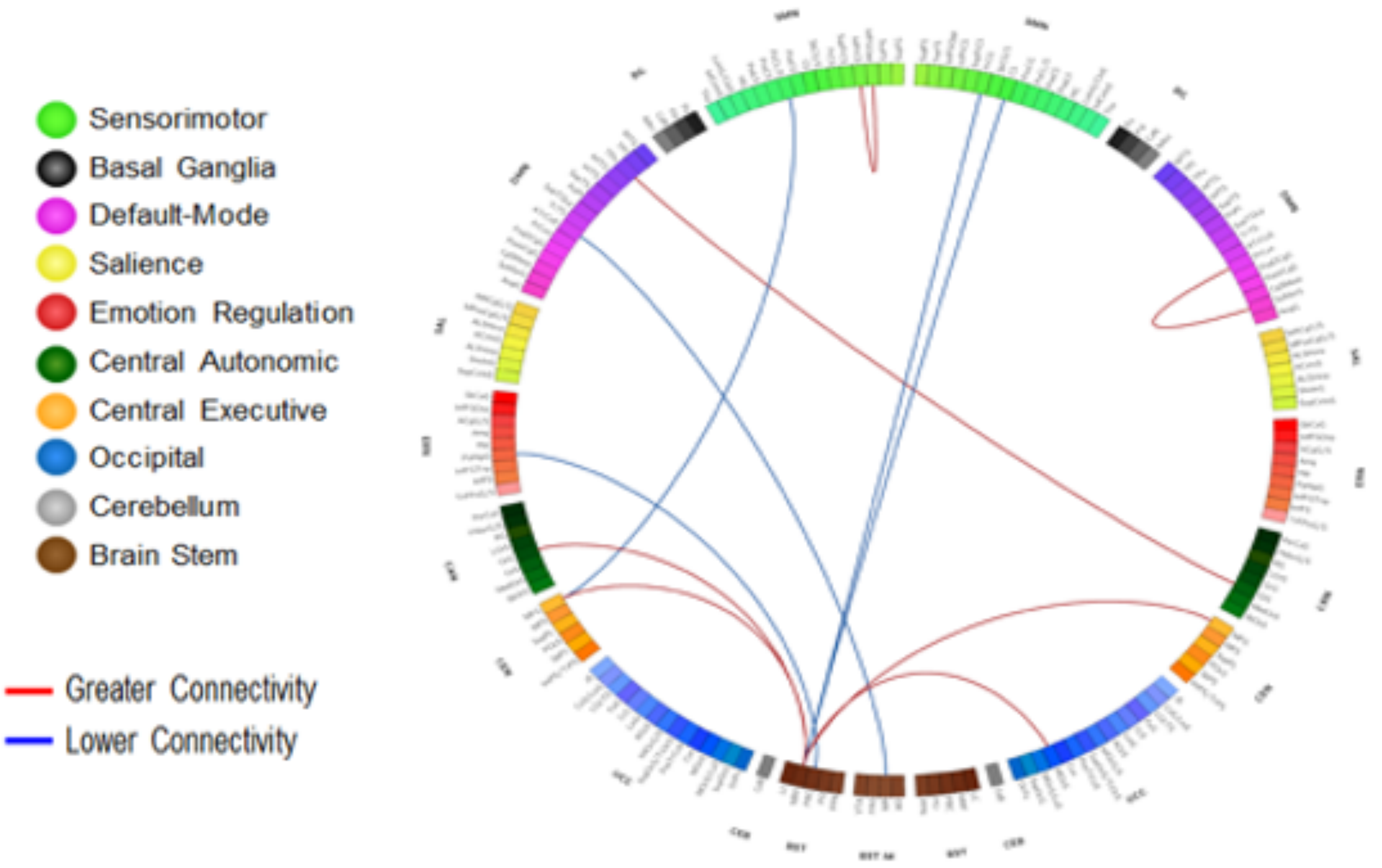

Supplement: Supplementary file 1 — Supplementary Information 1. [file 41598_2021_83116_MOESM1_ESM.pdf]
